# NAC transcription factors in plant multiple abiotic stress responses: progress and prospects

**DOI:** 10.3389/fpls.2015.00902

**Published:** 2015-10-29

**Authors:** Hongbo Shao, Hongyan Wang, Xiaoli Tang

**Affiliations:** ^1^Jiangsu Key Laboratory for Bioresources of Saline Soils; Provincial Key Laboratory of Agrobiology, Institute of Agro-biotechnology, Jiangsu Academy of Agricultural SciencesNanjing, China; ^2^Key Laboratory of Coastal Biology and Bioresources, Yantai Institute of Coastal Zone Research (YIC), Chinese Academy of Sciences (CAS)Yantai, China; ^3^Institute of Technology, Yantai Academy of China Agriculture UniversityYantai, China

**Keywords:** abiotic stress, multiple stresses, NAC, transcription factors, transgenic plant

## Abstract

Abiotic stresses adversely affect plant growth and agricultural productivity. According to the current climate prediction models, crop plants will face a greater number of environmental stresses, which are likely to occur simultaneously in the future. So it is very urgent to breed broad-spectrum tolerant crops in order to meet an increasing demand for food productivity due to global population increase. As one of the largest families of transcription factors (TFs) in plants, NAC TFs play vital roles in regulating plant growth and development processes including abiotic stress responses. Lots of studies indicated that many stress-responsive NAC TFs had been used to improve stress tolerance in crop plants by genetic engineering. In this review, the recent progress in NAC TFs was summarized, and the potential utilization of NAC TFs in breeding abiotic stress tolerant transgenic crops was also be discussed. In view of the complexity of field conditions and the specificity in multiple stress responses, we suggest that the NAC TFs commonly induced by multiple stresses should be promising candidates to produce plants with enhanced multiple stress tolerance. Furthermore, the field evaluation of transgenic crops harboring *NAC* genes, as well as the suitable promoters for minimizing the negative effects caused by over-expressing some *NAC* genes, should be considered.

## Introduction

As sessile organisms, plants continuously suffer from a broad range of environmental stresses including abiotic and biotic stresses. Abiotic stresses such as drought, salinity, heat and cold, adversely affect plant growth and agriculture productivity, and cause more than 50% of worldwide yield loss for major crops every year (Boyer, 1982; Bray et al., 2000; Shao et al., 2009; Ahuja et al., 2010; Lobell et al., 2011). Further to this, plants are also attacked by a vast range of pests and pathogens, including fungi, bacteria, viruses, nematodes, and herbivorous insects (Hammond-Kosack and Jones, 2000). In addition, current climate prediction models indicate the deterioration of climate including an increasing average temperature, a changing distribution of annual precipitation, a rise of sea level, and so on. This will be concurrent with an increased frequency of drought, flood, heat wave, and salinization (Easterling et al., 2000; IPCC, 2007, 2008; Mittler and Blumwald, 2010). Climate change will also affect the spread of pests and pathogens. For example, the increasing temperature can facilitate pathogen spread (Bale et al., 2002; Luck et al., 2011; Nicol et al., 2011), and many abiotic stress can weaken the defense mechanism of plants and increase their susceptibility to pathogen infection (Amtmann et al., 2008; Goel et al., 2008; Mittler and Blumwald, 2010; Atkinson and Urwin, 2012). Taken together, crop plants will face a greater range and number of environmental stresses, which are likely to occur simultaneously. So it is very urgent to breed stress-tolerant crop varieties to satisfy an increasing demand for food productivity due to global population increase (Takeda and Matsuoka, 2008; Newton et al., 2011).

To cope with these recurrent environmental stresses, plants can activate a number of defense mechanisms which include signal perception, signal transduction through either ABA-dependent or ABA-independent pathways, stress-responsive gene expression, in turn the activation of physiological and metabolic responses (Xiong et al., 2002; Chaves et al., 2003; Yamaguchi-Shinozaki and Shinozaki, 2006; Perez-Clemente et al., 2013). To date, a large array of stress responsive genes have been identified in many plants, including *Arabidopsis* and rice. These genes are generally classified into two types (Shinozaki et al., 2003). One is functional genes encoding important enzymes and metabolic proteins (functional proteins), such as detoxification enzyme, water channel, late embryogenesis abundant (LEA) protein, which directly function to protect cells from stresses. The other is regulatory genes encoding various regulatory proteins including transcription factors (TFs) and protein kinases, which regulate signal transduction and gene expression in the stress response. In the signal transduction processes, TFs play pivotal roles in the conversion of stress signal perception to stress-responsive gene expression. TFs and their interacting cis-elements function in the promoter region of different stress-related genes acting as molecular switches for gene expression. In plants ~7% of the genome encodes for putative TFs, which often belong to large gene families, such as *WRKY, bZIP, MYB, AP2/EREBP*, and *NAC* families (Udvardi et al., 2007; Golldack et al., 2011). In light of the key importance of TFs in controlling a wide range of downstream events, lots of studies have aimed to identify and characterize various TFs involved in stress responses. However, these studies have mostly focused on understanding the responses of model plants and crops to a single stress such as drought, salinity, heat or cold, pathogen infection, and so on (Hirayama and Shinozaki, 2010; Chew and Halliday, 2011). Unlike the controlled conditions in the laboratory, crops and other plants are often simultaneously subjected to multiple stresses in the field conditions (Ahuja et al., 2010). Recent studies have showed that plant response to a combination of drought and heat is not a simple additive effect of the individual stress, and the combination of multiple stresses produces a unique pattern of gene expression, which is distinct from the study of either stress individually (Rizhsky et al., 2002, 2004; Prasch and Sonnewald, 2013; Rasmussen et al., 2013). Therefore, the results of studies performed under individual stress factors are not suitable for the complex field conditions, and it is crucial to characterize the response of plants to multiple stresses and identify multiple stress responsive genes by imposing multiple stresses simultaneously as an entirely new stress (Mittler, 2006). Maybe, manipulation of these multiple stress responsive genes, especially multifunctional TFs, will provide the opportunity to breed the broad-spectrum tolerant crops with high yields. Based on these considerations above, this paper reviews the progress of NAC TFs involved in plant abiotic stress responses, and also prospects the future study direction for the challenge of multiple environmental stresses in agriculture, particularly concerning their potential utilization for plant multiple stress tolerance in the field conditions.

## NAC transcription factors in plants

As one of the largest family of TFs in plants, the NAC TFs comprise a complex plant-specific superfamily and are present in a wide range of species. The NAC acronym is derived from three earliest characterized proteins with a particular domain (NAC domain) from petunia NAM (no apical meristem), *Arabidopsis* ATAF1/2 and CUC2 (cup-shaped cotyledon; Souer et al., 1996; Aida et al., 1997). By the availability of an ever-increasing number of complete plant genomes and EST sequences, large numbers of putative *NAC* genes have been identified in many sequenced species at genome-wide scale (As shown in Table 1), such as 117 in *Arabidopsis*, 151 in rice, 74 in grape, 152 in soybean, 204 in Chinese cabbage, 152 in maize, and so on. The large size of *NAC* family inevitably complicates the unraveling of their regulatory process.

**Table 1:** ***NAC* family in various plant species**.

**Species**	**Number of *NAC* family**	**References**
*Arabidopsis thaliana*	117	Nuruzzaman et al., 2010
Rice (*Oryza sativa*)	151	Nuruzzaman et al., 2010
Grape (*Vitis vinifera*)	74	Wang et al., 2013
Soybean (*Glycine max*)	152	Le et al., 2011
Pigeonpea (*Cajanus cajan*)	88	Satheesh et al., 2014
Foxtail millet (*Setaria italica L.*)	147	Puranik et al., 2013
Chinese cabbage (*Brassica rapa*)	204	Liu et al., 2014
*Brachypodium distachyon*	101	You et al., 2015
Physic Nut (*Jatropha curcas L.*)	100	Wu et al., 2015
Maize (*Zea mays*)	152	Shiriga et al., 2014
Apple (*Malus domestica)*	180	Su et al., 2013
Chickpea (*Cicer arietinum L.*)	71	Ha et al., 2014
Potato (*Solanum tuberosum*)	110	Singh et al., 2013
Poplar (*Populus trichocarpa*)	163	Hu et al., 2010
Banana (*Musa acuminata*)	167	Cenci et al., 2014
Tobacco (*Nicotiana tabacum*)	152	Rushton et al., 2008
Tomato (*Solanum lycopersicum*)	104	Su et al., 2015
Cassava (*Manihot esculenta Crantz*)	96	Hu et al., 2015
*Gossypium raimondii*	145	Shang et al., 2013

The *NAC* family has been found to function in various processes including shoot apical meristem (Takada et al., 2001), flower development (Sablowski and Meyerowitz, 1998), cell division (Kim et al., 2006), leaf senescence (Breeze et al., 2011), formation of secondary walls (Zhong et al., 2010), and biotic and abiotic stress responses (Olsen et al., 2005; Christianson et al., 2010; Tran et al., 2010; Nakashima et al., 2012). Nonetheless, only a few of these genes have been characterized to date and most of the *NAC* family members have not yet been studied, even though these genes are likely to play important roles in plants, and a great deal of work will be required to determine the specific biological function of each *NAC* gene. The intensive study on model plants including *Arabidopsis* and rice reveals that a typical NAC protein contains a highly conserved N-terminal DNA-binding NAC domain and a variable transcriptional regulatory region in the C-terminal region. The NAC domain with ~150–160 amino acids is divided into five sub-domains (A to E; Ooka et al., 2003). The function of the NAC domain has been associated with nuclear localization, DNA binding, and the formation of homodimers or heterodimers with other NAC domain-containing proteins (Olsen et al., 2005). In contrast, the highly diverged C-terminal region functions as a transcription regulatory region, acting as a transcriptional activator or repressor, but it has frequent occurrence of simple amino acid repeats and regions rich in serine and threonine, proline and glutamine, or acidic residues (Olsen et al., 2005; Puranik et al., 2012). Some NAC TFs also contain transmembrane motifs in the C-terminal region which are responsible for anchoring to plasma membrane or endoplasmic reticulum, and these NAC TFs are membrane-associated and designated as NTLs (Seo et al., 2008; Seo and Park, 2010).

The expression of *NAC* genes can firstly be regulated at the level of transcription because there are some stress-responsive cis-acting elements contained in the promoter region such as *ABREs* (ABA-responsive elements), *DREs* (Dehydration-responsive elements), jasmonic acid responsive element and salicylic acid responsive element. Then the complex post-transcriptional regulation involves microRNA-mediated cleavage of genes or alternative splicing. *NAC* TFs also undergo intensive post-translational regulation including ubiquitinization, dimerization, phosphorylation or proteolysis (Nakashima et al., 2012; Puranik et al., 2012). These regulatory steps help NAC TFs playing multiple roles in the majority of plant processes as mentioned above. The NAC TFs regulate the transcription of downstream target genes by binding to a consensus sequence in their promoters. The *NAC* recognition sequence (NACRS) containing the CACG core-DNA binding motif has been identified in the promoter of the drought inducible EARLY RESPONSE TO DEHYDRATION1 (*ERD1*) gene in *Arabidopsis* (Simpson et al., 2003; Tran et al., 2004). The rice drought-inducible ONAC TFs also can bind to a similar NACRS, demonstrating that the *NACRS* might be conserved across plants at least for stress-inducible NAC TFs (Hu et al., 2006; Fang et al., 2008). In addition, other sequences have also been reported as NAC binding sites (*NACBS*), such as an *Arabidopsis* calmodulin-binding NAC with GCTT as core-binding motif (Kim et al., 2007), the iron deficiency-responsive *IDE2* motif containing the core sequence CA(A/C)G(T/C) (T/C/A) (T/C/A) (Ogo et al., 2008) and the secondary wall NAC binding element (SNBE) with (T/A)NN(C/T) (T/C/G)TNNNNNNNA(A/C)GN(A/C/T) (A/T) as consensus sequence (Zhong et al., 2010). The sequences flanking the core site in promoter of target genes may define the binding specificity of different NAC TFs. Thus, the NAC TF family can recognize a vast array of DNA-Binding sequences and regulate multiple downstream target genes. These target genes regulated by NAC TFs comprise regulatory genes encoding regulatory proteins which function in signal transduction and regulation of gene expression and functional genes encoding proteins which are involved in osmolyte production, reactive oxygen species scavenging and detoxification, macromolecule protection and ubiquitination (Puranik et al., 2012). Taken together, the existence of NACRS in promoter of some of these genes makes them to be the potential direct targets, whereas those that do not have this motif may not be direct targets. In future more other novel NACRS remain to be elucidated by microarrays combined with chromatin immunoprecipitation (Taverner et al., 2004).

## NAC transcription factors function in abiotic stress

The NAC TFs play a vital role in the complex signaling networks during plant stress responses. Because of the large number of NAC TFs from different plants and their unknown roles, it is still a great challenge to uncover their roles in abiotic stress. Recently, whole-genome expression profiling and transcriptome studies have enabled researchers to identify a number of putative NAC TFs involved in abiotic stress responses. For example, 33 *NAC* genes changed significantly in *Arabdopsis* under salt treatment (Jiang and Deyholos, 2006), 38 *NAC* genes were involved in response to drought in soybean (Le et al., 2011), 40 *NAC* genes responded to drought or salt stress in rice (Fang et al., 2008), 32 *NAC* genes responded to at least two kinds of treatments in *Chrysanthemum lavandulifolium* (Huang et al., 2012). It appears that a significant proportion of *NAC* genes function in stress response according to the expression data from genome-wide transcriptome analyses in many plants. Phylogenetic analyses of NAC TFs showed that most of the stress responsive NAC TFs appeared to contain a closely homologous NAC domain (Ernst et al., 2004; Fang et al., 2008). Moreover, the stress-responsive *NAC* genes exhibit a large diversity in expression patterns, indicating their involvement in the regulation of a wide spectrum of responses to different abiotic stresses. The precise regulations of *NAC* genes during plant abiotic stress responses contribute to the establishment of complex signaling networks, and the important roles of *NAC* genes in plant abiotic stress responses make them promising candidates for the generation of stress tolerant transgenic plants. The functional studies of NAC TFs by over-expression techniques will directly improve our understanding of the regulatory functions of NAC members to abiotic stresses. Transgenic constructs over-expressing the selected *NAC* genes have been made in *Arabidopsis*, rice and other plants. Some successful examples are summarized in Table 2.

**Table 2:** **Abiotic stress tolerance of transgenic plant over-expressing *NAC* genes**.

**Transgenic plant**	**Genotype**	**Enhanced tolerance**	**References**
*A. thaliana*	*ANAC019* overexpression	Drought, high-salinity, ABA signaling	Tran et al., 2004
	*ANAC055* overexpression	Drought, high-salinity, ABA signaling	Tran et al., 2004
	*ANAC72* overexpression	Drought, high-salinity, ABA signaling	Tran et al., 2004
	*RD26* overexpression	Drought, salt, ABA signaling	Fujita et al., 2004
	*ANAC019* overexpression	Cold, ABA signaling	Jensen et al., 2010
	*ATAF1* overexpression	Positive regulator of drought tolerance	Wu et al., 2009
	*ONAC063* overexpression	Higher seed germination under high salinity and osmotic stress	Yokotani et al., 2009
	*GmNAC20* overexpression	Salt and freezing tolerance	Hao et al., 2011
	*ZmSNAC1* overexpression	Low temperature, high-salinity, drought, and ABA signaling	Lu et al., 2012
	*TaNAC2* overexpression	Drought, salt, and freezing stresses	Mao et al., 2012
	*ANAC042* overexpression	Heat stress	Shahnejat-Bushehri et al., 2012
*O. sativa*	*SNAC1* overexpression	Increased stomatal closure and drought resistance in dry field conditions, salt toleranc	Hu et al., 2006
	*SNAC2* overexpression	Salt, drought, disease resistance drought, salinity, cold, wounding, and ABA treatment	Sindhu et al., 2008
	*OsNAC4* overexpression	Drought, salt, cold tolerance	Zheng et al., 2009
	*OsNAC5* overexpression	ABA, salt, cold tolerance, grain filling	Sperotto et al., 2009
	*OsNAC6* overexpression	Drought and salt tolerance	Nakashima et al., 2007
	*ONAC10* overexpression	Drought, high salinity, low temperature toleranc	Jeong et al., 2010
	*ONAC045* overexpression	Drought and salt tolerance	Song et al., 2011
*N. tabacum*	*TaNAC2a* overexpression	Drought tolerance	Tang et al., 2012
	*DgNAC1* overexpression	ABA, NaCl, drought, and cold	Liu et al., 2011a
	*EcNAC1* overexpression	Water-deficit and salt stress	Ramegowda et al., 2012
*T. aestivum*	*TaNAC69* overexpression	PEG-induced dehydration and mild salt tolerance	Xue et al., 2011
*G. max*	*GmNAC11* overexpression	Salt tolerance in soybean transgenic hairy roots	Hao et al., 2011

## Conclusions and perspectives

Considerable information has been gained about NAC TFs since the discovery of NAC TFs, but the research in this area is still in its infancy. Genome-wide identification and expression profiling will undoubtedly open new avenues for describing the key features of NAC TFs. As a result, our current understandings of the regulatory functions of the NAC TFs in various plant species will be definitely accelerated. In particular, the stress-responsive *NAC* TFs can be used as promising candidates for generation of stress tolerant transgenic plants possessing high productivity under adverse conditions. As a matter of fact, many transgenic studies have been proved successful by gene manipulation of *NAC* TFs for conferring different stresses tolerance to plants (As shown in Table 2), but there are still some problems to be solved. Firstly, the constitutive overexpression of *NAC* genes occasionally may lead to negative effects in transgenic plants such as dwarfing, late flowering and lower yields (Fujita et al., 2004; Nakashima et al., 2007; Hao et al., 2011; Liu et al., 2011b). Secondly, the transgenic plants overexpressing *NAC* genes may occasionally have antagonistic responses to different stresses. For example, drought tolerant *Arabidopsis* plants overexpressing *ATAF1* were highly sensitive to ABA, high-salt, oxidative stress and necrotrophic fungus (*B. cinerea*; Wu et al., 2009). Overexpressing *ANAC019* and *ANAC055* not only increased drought tolerance but also decreased resistance to *B. cinerea* (Fujita et al., 2004; Bu et al., 2008). Thirdly, only a few of transgenic plants overexpressing *NAC* genes were evaluated in the field trials so far, and most of them were tested in greenhouse conditions and focused on plant vegetative stages rather than reproductive stages (Valliyodan and Nguyen, 2006). Lastly, most of the studies on *NAC* TFs only investigated the molecular mechanisms of individual occurring stress situations. Although recent studies have conducted multi-parallel stress experiments and identified different *NAC* TFs responding to single stress situations (Huang et al., 2012), the knowledge concerning responses to combinations of several stress factors is scarce, especially interactions among stress factors.

**Figure 1:**
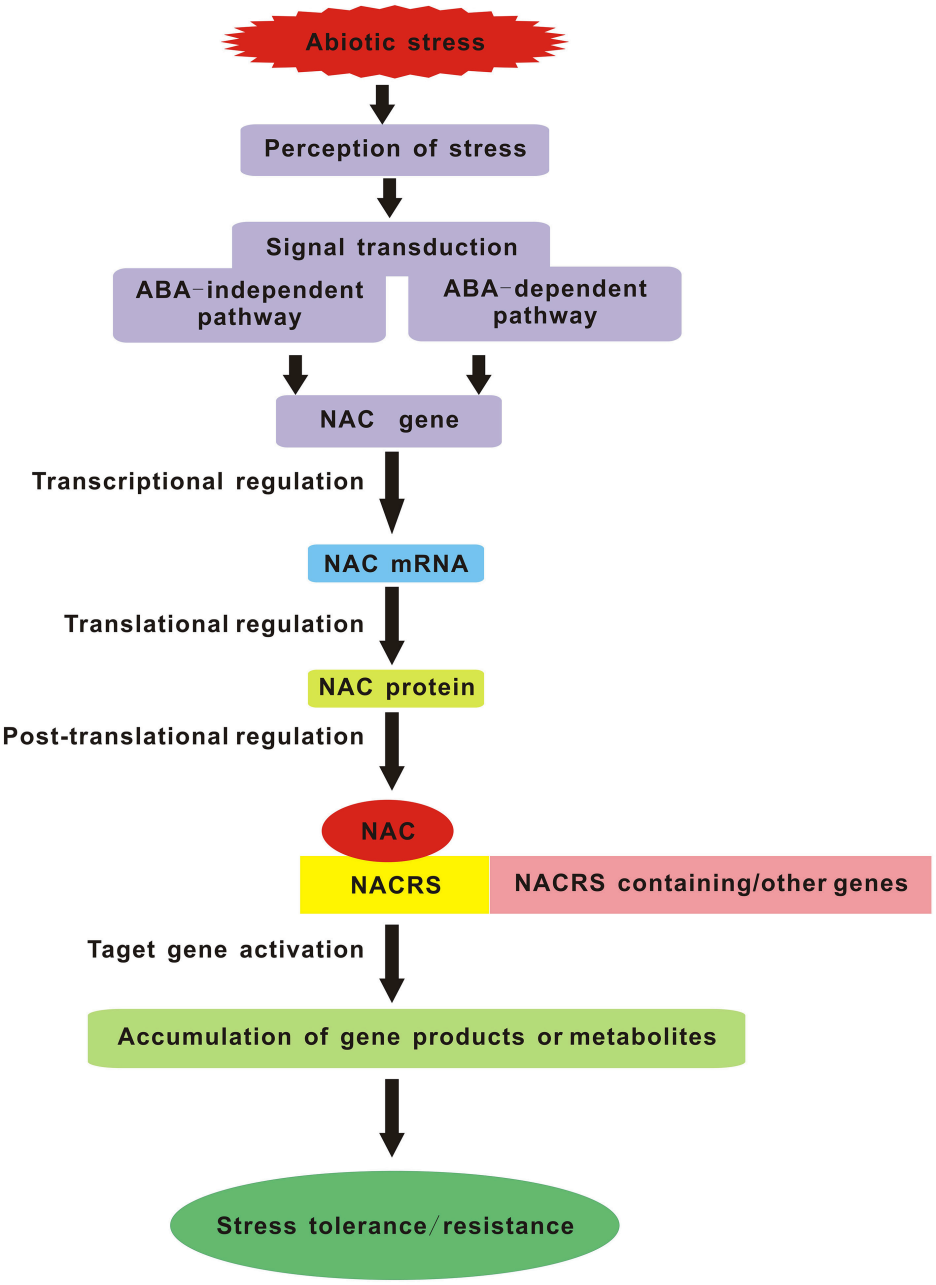
**Schematic diagram of NAC TFs as key components in transcriptional regulatory networks during abiotic stress**. NACRS is NAC recognition sequence.

As everyone knows, one of the most important aims for plant stress research is to provide targets for the improvement of stress tolerance in crop plants. With the forecast changes in climatic conditions leading to a more complex stress environment in the fields, we will face new challenges in creating the multiple stress-tolerant crops. Breeding such plants will depend on understanding the crucial stress-regulatory networks and the potential effects of different combinations of adverse conditions. Studies of multiple stress responses in *Arabidopsis* have provided us with several possible avenues. Master regulatory genes such as members of the *MYC, MYB*, and *NAC* TF families that act in multiple abiotic stress responses are excellent candidates for manipulating multiple stress tolerance. So in the future, it is crucial to impose multiple stresses simultaneously that simulate natural field conditions and regard each set of stress combinations as an entirely new stress in order to identify the corresponding *NAC* TFs commonly induced by multiple stresses. Manipulation of these genes should be the major target of attempts to produce plants with enhanced multiple stress tolerance. Furthermore, the potential *NAC* genes which confer multiple abiotic stress tolerance in model plant species must be tested in crop plants and greater emphasis should be placed on the field evaluation of the transgenic crops harboring *NAC* genes, especially focusing on their reproductive success. Another lesson is the selection and/or improvement of suitable promoters (such as a stress-inducible promoter) which can maximize the positive effects and minimize the negative effects caused by over-expressing some *NAC* genes. In summary, NAC TFs are the key components of the signaling pathway in stress response which carry out their function by interacting with both downstream and upstream partners (Figure 1). Understanding the molecular mechanisms of NAC TFs networks integrating multiple stress responses will be essential for the development of broad-spectrum stress tolerant crop plants that can better cope with environmental challenges in future climates.

### Conflict of interest statement

The authors declare that the research was conducted in the absence of any commercial or financial relationships that could be construed as a potential conflict of interest.

## References

[B1] Ahuja I. de Vos R. C. Bones A. M. Hall R. D. (2010). Plant molecular stress responses face climate change. Trends Plant Sci. 15, 664–674. 10.1016/j.tplants.2010.08.002 20846898

[B2] Aida M. Ishida T. Fukaki H. Fujisawa H. Tasaka M. (1997). Genes involved in organ separation in *Arabidopsis*: an analysis of the cup-shaped cotyledon mutant. Plant Cell 9, 841–857. 10.1105/tpc.9.6.841 9212461 PMC156962

[B3] Amtmann A. Troufflard S. Armengaud P. (2008). The effect of potassium nutrition on pest and disease resistance in plants. Physiol. Plant. 133, 682–691. 10.1111/j.1399-3054.2008.01075.x 18331404

[B4] Atkinson N. J. Urwin P. E. (2012). The interaction of plant biotic and abiotic stresses: from genes to the field. J. Exp. Bot. 63, 3523–3543. 10.1093/jxb/ers100 22467407

[B5] Bale J. S. Masters G. J. Hodkinson I. D. Awmack C. Bezemer T. M. Brown V. K. (2002). Herbivory in global climate change research: direct effects of rising temperature on insect herbivores. Glob. Change Biol. 8, 1–16. 10.1046/j.1365-2486.2002.00451.x

[B6] Boyer J. S. (1982). Plant productivity and environment. Science 218, 443–448. 10.1126/science.218.4571.443 17808529

[B7] Bray E. A. Bailey-Serres J. Weretilnyk E. (2000). Responses to abiotic stresses, in Biochemistry and Molecular Biology of Plants, eds Gruissem W. Buchannan B. Jones R. (Rockville, MD: American Society of Plant Physiologists), 1158–1249.

[B8] Breeze E. Harrison E. McHattie S. Hughes L. Hickman R. Hill C.. (2011). High-resolution temporal profiling of transcripts during *Arabidopsis* leaf senescence reveals a distinct chronology of processes and regulation. Plant Cell 23, 873–894. 10.1105/tpc.111.083345 21447789 PMC3082270

[B9] Bu Q. Jiang H. Li C. B. Zhai Q. Zhang J. Wu X.. (2008). Role of the *Arabidopsis thaliana* NAC transcription factors ANAC019 and ANAC055 in regulating jasmonic acid-signaled defense responses. Cell Res. 18, 756–767. 10.1038/cr.2008.53 18427573

[B10] Cenci A. Guignon V. Roux N. Rouard M. (2014). Genomic analysis of NAC transcription factors in banana (*Musa acuminata*) and definition of NAC orthologous groups for monocots and dicots. Plant Mol. Biol. 85, 63–80. 10.1007/s11103-013-0169-2 24570169 PMC4151281

[B11] Chaves M. M. Maroco J. Joao S. P. (2003). Understanding plant responses to drought - from genes to the whole plant. Funct. Plant Biol. 30, 239–264. 10.1071/FP02076 32689007

[B12] Chew Y. H. Halliday K. J. (2011). A stress-free walk from *Arabidopsis* to crops. Curr. Opin. Biotechnol. 22, 281–286. 10.1016/j.copbio.2010.11.011 21168324

[B13] Christianson J. A. Dennis E. S. Llewellyn D. J. Wilson I. W. (2010). ATAF NAC transcription factors: regulators of plant stress signaling. Plant Signal. Behav. 5, 428–432. 10.4161/psb.5.4.10847 20118664 PMC7080423

[B14] Easterling D. R. Meehl G. A. Parmesan C. Changnon S. A. Karl T. R. Mearns L. O. (2000). Climate extremes: observations, modeling, and impacts. Science 289, 2068–2074. 10.1126/science.289.5487.2068 11000103

[B15] Ernst H. A. Olsen A. N. Larsen S. Lo Leggio L. (2004). Structure of the conserved domain of ANAC, a member of the NAC family of transcription factors. EMBO Rep. 5, 297–303. 10.1038/sj.embor.7400093 15083810 PMC1299004

[B16] Fang Y. You J. Xie K. Xie W. Xiong L. (2008). Systematic sequence analysis and identification of tissue-specific or stress-responsive genes of NAC transcription factor family in rice. Mol. Genet. Genomics 280, 547–563. 10.1007/s00438-008-0386-6 18813954

[B17] Fujita M. Fujita Y. Maruyama K. Seki M. Hiratsu K. Ohme-Takagi M.. (2004). A dehydration-induced NAC protein, RD26, is involved in a novel ABA-dependent stress-signaling pathway. Plant J. 39, 863–876. 10.1111/j.1365-313X.2004.02171.x 15341629

[B18] Goel A. K. Lundberg D. Torres M. A. Matthews R. Akimoto-Tomiyama C. Farmer L.. (2008). The *Pseudomonas syringae* type III effector HopAM1 enhances virulence on water-stressed plants. Mol. Plant-Microbe Interact. 21, 361–370. 10.1094/MPMI-21-3-0361 18257685

[B19] Golldack D. Lüking I. Yang O. (2011). Plant tolerance to drought and salinity: stress regulating transcription factors and their functional significance in the cellular transcriptional network. Plant Cell Rep. 30, 1383–1391. 10.1007/s00299-011-1068-0 21476089

[B20] Ha C. V. Esfahani M. N. Watanabe Y. Tran U. T. Sulieman S. Mochida K.. (2014). Genome-wide identification and expression analysis of the CaNAC family members in chickpea during development, dehydration and ABA treatments. PLoS ONE 9:e114107. 10.1371/journal.pone.0114107 25479253 PMC4257607

[B21] Hammond-Kosack K. E. Jones J. D. G. (2000). Response to plant pathogens, in Biochemistry and Molecular Biology of Plants, eds Buchannan B. Gruissem W. Jones R. (Rockville, MD: American Society of Plant Physiologists), 1102–1157.

[B22] Hao Y. J. Wei W. Song Q. X. Chen H. W. Zhang Y. Q. Wang F.. (2011). Soybean NAC transcription factors promote abiotic stress tolerance and lateral root formation in transgenic plants. Plant J. 68, 302–313. 10.1111/j.1365-313X.2011.04687.x 21707801

[B23] Hirayama T. Shinozaki K. (2010). Research on plant abiotic stress responses in the post-genome era: past, present and future. Plant J. 61, 1041–1052. 10.1111/j.1365-313X.2010.04124.x 20409277

[B24] Hu H. Dai M. Yao J. Xiao B. Li X. Zhang Q.. (2006). Overexpressing a NAM, ATAF, and CUC (NAC) transcription factor enhances drought resistance and salt tolerance in rice. Proc. Natl. Acad. Sci. U.S.A. 103, 12987–12992. 10.1073/pnas.0604882103 16924117 PMC1559740

[B25] Hu R. Qi G. Kong Y. Kong D. Gao Q. Zhou G. (2010). Comprehensive analysis of NAC domain transcription factor gene family in *Populus trichocarpa*. BMC Plant Biol. 10:145. 10.1186/1471-2229-10-145 20630103 PMC3017804

[B26] Hu W. Wei Y. Xia Z. Yan Y. Hou X. Zou M.. (2015). Genome-wide identification and expression analysis of the NAC transcription factor family in cassava. PLoS ONE 10:e0136993. 10.1371/journal.pone.0136993 26317631 PMC4552662

[B27] Huang H. Wang Y. Wang S. Wu X. Yang K. Niu Y.. (2012). Transcriptome-wide survey and expression analysis of stress-responsive NAC genes in *Chrysanthemum lavandulifolium*. Plant Sci. 193–194, 18–27. 10.1016/j.plantsci.2012.05.004 22794915

[B28] IPCC (2007). Climate change 2007: the physical science basis, in Contribution of Working Group I to the Fourth Assessment Report of the Intergovernmental Panel on Climate Change, eds Solomon S. Qin D. Manning M. Chen Z. Marquis M. Averyt K. B. (Geneva: IPCC Secretariat).

[B29] IPCC (2008). Climate change and water, in Technical Paper of the Intergovernmental Panel on Climate Change, eds Bates B. C. Kundzewicz Z. W. Palutikof J. Wu S. (Geneva: IPCC Secretariat).

[B30] Jensen M. K. Kjaersgaard T. Nielsen M. M. Galberg P. Petersen K. O'shea C.. (2010). The *Arabidopsis thaliana* NAC transcription factor family: structure-function relationships and determinants of ANAC019 stress signalling. Biochem. J. 426, 183–196. 10.1042/BJ20091234 19995345

[B31] Jeong J. S. Kim Y. S. Baek K. H. Jung H. Ha S. H. Do Choi Y.. (2010). Root-specific expression of OsNAC10 improves drought tolerance and grain yield in rice under field drought conditions. Plant Physiol. 153, 185–197. 10.1104/pp.110.154773 20335401 PMC2862432

[B32] Jiang Y. Deyholos M. K. (2006). Comprehensive transcriptional profiling of NaCl-stressed *Arabidopsis* roots reveals novel classes of responsive genes. BMC Plant Biol. 6:25. 10.1186/1471-2229-6-25 17038189 PMC1621065

[B33] Kim H. S. Park B. O. Yoo J. H. Jung M. S. Lee S. M. Han H. J.. (2007). Identification of a calmodulin-binding NAC protein as a transcriptional repressor in *Arabidopsis*. J. Biol. Chem. 282, 36292–36302. 10.1074/jbc.M705217200 17947243

[B34] Kim Y. S. Kim S. G. Park J. E. Park H. Y. Lim M. H. Chua N. H.. (2006). A membrane-bound NAC transcription factor regulates cell division in *Arabidopsis*. Plant Cell 18, 3132–3144. 10.1105/tpc.106.043018 17098812 PMC1693948

[B35] Le D. T. Nishiyama R. Watanabe Y. Mochida K. Yamaguchi-Shinozaki K. Shinozaki K.. (2011). Genome-wide survey and expression analysis of the plant-specific NAC transcription factor family in soybean during development and dehydration stress. DNA Res. 18, 263–276. 10.1093/dnares/dsr015 21685489 PMC3158466

[B36] Liu Q. L. Xu K. D. Zhao L. J. Pan Y. Z. Jiang B. B. Zhang H. Q.. (2011a). Overexpression of a novel chrysanthemum NAC transcription factor gene enhances salt tolerance in tobacco. Biotechnol. Lett. 33, 2073–2082. 10.1007/s10529-011-0659-8 21660574

[B37] Liu T. K. Song X. M. Duan W. K. Huang Z. N. Liu G. F. Li Y. (2014). Genome-wide analysis and expression patterns of NAC transcription factor family under different developmental stages and abiotic stresses in Chinese cabbage. Plant Mol. Biol. Rep. 32, 1041–1056. 10.1007/s11105-014-0712-6

[B38] Liu X. Hong L. Li X. Y. Yao Y. Hu B. Li L. (2011b). Improved drought and salt tolerance in transgenic *Arabidopsis* overexpressing a NAC transcriptional factor from *Arachis hypogaea*. Biosci. Biotechnol. Biochem. 75, 443–450. 10.1271/bbb.100614 21389632

[B39] Lobell D. B. Schlenker W. Costa-Roberts J. (2011). Climate trends and global crop production since 1980. Science 333, 616–620. 10.1126/science.1204531 21551030

[B40] Luck J. Spackman M. Freeman A. Trębicki P. Griffiths W. Finlay K. (2011). Climate change and diseases of food crops. Plant Pathol. 60, 113–121. 10.1111/j.1365-3059.2010.02414.x

[B41] Lu M. Ying S. Zhang D. F. Shi Y. S. Song Y. C. Wang T. Y.. (2012). A maize stress-responsive NAC transcription factor, ZmSNAC1, confers enhanced tolerance to dehydration in transgenic *Arabidopsis*. Plant Cell Rep. 31, 1701–1711. 10.1007/s00299-012-1284-2 22610487

[B42] Mao X. Zhang H. Qian X. Li A. Zhao G. Jing R. (2012). TaNAC2, a NAC-type wheat transcription factor conferring enhanced multiple abiotic stress tolerances in *Arabidopsis*. J. Exp. Bot. 63, 2933–2946. 10.1093/jxb/err462 22330896 PMC3350912

[B43] Mittler R. (2006). Abiotic stress, the field environment and stress combination. Trends Plant Sci. 11, 15–19. 10.1016/j.tplants.2005.11.002 16359910

[B44] Mittler R. Blumwald E. (2010). Genetic engineering for modern agriculture: challenges and perspectives. Annu. Rev. Plant Biol. 61, 443–462. 10.1146/annurev-arplant-042809-112116 20192746

[B45] Nakashima K. Takasaki H. Mizoi J. Shinozaki K. Yamaguchi-Shinozaki K. (2012). NAC transcription factors in plant abiotic stress responses. Biochim. Biophys. Acta 1819, 97–103. 10.1016/j.bbagrm.2011.10.005 22037288

[B46] Nakashima K. Tran L. S. van Nguyen D. Fujita M. Maruyama K. Todaka D.. (2007). Functional analysis of a NAC-type transcription factor OsNAC6 involved in abiotic and biotic stress-responsive gene expression in rice. Plant J. 51, 617–630. 10.1111/j.1365-313X.2007.03168.x 17587305

[B47] Newton A. C. Johnson S. N. Gregory P. J. (2011). Implications of climate change for diseases, crop yields and food security. Euphytica 179, 3–18. 10.1007/s10681-011-0359-4

[B48] Nicol J. M. Turner S. J. Coyne D. L. Nijs L. D. Hockland S. Maafi Z. T. (2011). Current nematode threats to world agriculture, in Genomics and Molecular Genetics of Plant-Nematode Interactions, eds Jones J. Gheysen G. Fenoll C. (Berlin: Springer), 21–43. 10.1007/978-94-007-0434-3_2

[B49] Nuruzzaman M. Manimekalai R. Sharoni A. M. Satoh K. Kondoh H. Ooka H.. (2010). Genome-wide analysis of NAC transcription factor family in rice. Gene 465, 30–44. 10.1016/j.gene.2010.06.008 20600702

[B50] Ogo Y. Kobayashi T. Nakanishi Itai R. Nakanishi H. Kakei Y. Takahashi M.. (2008). A novel NAC transcription factor, IDEF2, that recognizes the iron deficiency-responsive element 2 regulates the genes involved in iron homeostasis in plants. J. Biol. Chem. 283, 13407–13417. 10.1074/jbc.M708732200 18308732

[B51] Olsen A. N. Ernst H. A. Leggio L. L. Skriver K. (2005). NAC transcription factors: structurally distinct, functionally diverse. Trends Plant Sci. 10, 79–87. 10.1016/j.tplants.2004.12.010 15708345

[B52] Ooka H. Satoh K. Doi K. Nagata T. Otomo Y. Murakami K.. (2003). Comprehensive analysis of NAC family genes in *Oryza sativa* and *Arabidopsis thaliana*. DNA Res. 10, 239–247. 10.1093/dnares/10.6.239 15029955

[B53] Pérez-Clemente R. M. Vives V. Zandalinas S. I. López-Climent M. F. Muñoz V. Gómez-Cadenas A. (2013). Biotechnological approaches to study plant responses to stress. Biomed Res. Int. 2013:654120. 10.1155/2013/654120 23509757 PMC3591138

[B54] Prasch C. M. Sonnewald U. (2013). Simultaneous application of heat, drought, and virus to *Arabidopsis* plants reveals significant shifts in signaling networks. Plant Physiol. 162, 1849–1866. 10.1104/pp.113.221044 23753177 PMC3729766

[B55] Puranik S. Sahu P. P. Mandal S. N. B V. S. Parida S. K. Prasad M. (2013). Comprehensive genome-wide survey, genomic constitution and expression profiling of the NAC transcription factor family in foxtail millet (*Setaria italica* L.). PLoS ONE 8:e64594. 10.1371/journal.pone.0064594 23691254 PMC3654982

[B56] Puranik S. Sahu P. P. Srivastava P. S. Prasad M. (2012). NAC proteins: regulation and role in stress tolerance. Trends Plant Sci. 17, 369–381. 10.1016/j.tplants.2012.02.004 22445067

[B57] Ramegowda V. Senthil-Kumar M. Nataraja K. N. Reddy M. K. Mysore K. S. Udayakumar M. (2012). Expression of a finger millet transcription factor, EcNAC1, in tobacco confers abiotic stress-tolerance. PLoS ONE 7:e40397. 10.1371/journal.pone.0040397 22808152 PMC3394802

[B58] Rasmussen S. Barah P. Suarez-Rodriguez M. C. Bressendorff S. Friis P. Costantino P.. (2013). Transcriptome responses to combinations of stresses in *Arabidopsis*. Plant Physiol. 161, 1783–1794. 10.1104/pp.112.210773 23447525 PMC3613455

[B59] Rizhsky L. Liang H. Mittler R. (2002). The combined effect of drought stress and heat shock on gene expression in tobacco. Plant Physiol. 130, 1143–1151. 10.1104/pp.006858 12427981 PMC166635

[B60] Rizhsky L. Liang H. Shuman J. Shulaev V. Davletova S. Mittler R. (2004). When defense pathways collide. The response of *Arabidopsis* to a combination of drought and heat stress. Plant Physiol. 134, 1683–1696. 10.1104/pp.103.033431 15047901 PMC419842

[B61] Rushton P. J. Bokowiec M. T. Han S. Zhang H. Brannock J. F. Chen X.. (2008). Tobacco transcription factors: novel insights into transcriptional regulation in the *Solanaceae*. Plant Physiol. 147, 280–295. 10.1104/pp.107.114041 18337489 PMC2330323

[B62] Sablowski R. W. Meyerowitz E. M. (1998). A homolog of NO APICAL MERISTEM is an immediate target of the floral homeotic genes APETALA3/PISTILLATA. Cell 92, 93–103. 10.1016/S0092-8674(00)80902-2 9489703

[B63] Satheesh V. Jagannadham P. T. Chidambaranathan P. Jain P. K. Srinivasan R. (2014). NAC transcription factor genes: genome-wide identification, phylogenetic, motif and cis-regulatory element analysis in pigeonpea (*Cajanus cajan* (L.) Millsp.). Mol. Biol. Rep. 41, 7763–7773. 10.1007/s11033-014-3669-5 25108674

[B64] Seo P. J. Kim S. G. Park C. M. (2008). Membrane-bound transcription factors in plants. Trends Plant Sci. 13, 550–556. 10.1016/j.tplants.2008.06.008 18722803

[B65] Seo P. J. Park C. M. (2010). A membrane-bound NAC transcription factor as an integrator of biotic and abiotic stress signals. Plant Signal. Behav. 5, 481–483. 10.4161/psb.11083 20139739 PMC7080469

[B66] Shahnejat-Bushehri S. Mueller-Roeber B. Balazadeh S. (2012). *Arabidopsis* NAC transcription factor JUNGBRUNNEN1 affects thermomemory-associated genes and enhances heat stress tolerance in primed and unprimed conditions. Plant Signal. Behav. 7, 1518–1521. 10.4161/psb.22092 23073024 PMC3578882

[B67] Shang H. Li W. Zou C. Yuan Y. (2013). Analyses of the NAC transcription factor gene family in *Gossypium raimondii* Ulbr.: chromosomal location, structure, phylogeny, and expression patterns. J. Integr. Plant Biol. 55, 663–676. 10.1111/jipb.12085 23756542

[B68] Shao H. B. Chu L. Y. Jaleel C. A. Manivannan P. Panneerselvam R. Shao M. A. (2009). Understanding water deficit stress-induced changes in the basic metabolism of higher plants - biotechnologically and sustainably improving agriculture and the ecoenvironment in arid regions of the globe. Crit. Rev. Biotechnol. 29, 131–151. 10.1080/07388550902869792 19412828

[B69] Shinozaki K. Yamaguchi-Shinozakiy K. Sekiz M. (2003). Regulatory network of gene expression in the drought and cold stress responses. Curr. Opin. Plant Biol. 6, 410–417. 10.1016/S1369-5266(03)00092-X 12972040

[B70] Shiriga K. Sharma R. Kumar K. Yadav S. K. Hossain F. Thirunavukkarasu N. (2014). Genome-wide identification and expression pattern of drought-responsive members of the NAC family in maize. Meta Gene 2, 407–417. 10.1016/j.mgene.2014.05.001 25606426 PMC4287890

[B71] Simpson S. D. Nakashima K. Narusaka Y. Seki M. Shinozaki K. Yamaguchi-Shinozaki K. (2003). Two different novel cis-acting elements of erd1, a clpA homologous *Arabidopsis* gene function in induction by dehydration stress and dark-induced senescence. Plant J. 33, 259–270. 10.1046/j.1365-313X.2003.01624.x 12535340

[B72] Sindhu A. Chintamanani S. Brandt A. S. Zanis M. Scofield S. R. Johal G. S. (2008). A guardian of grasses: specific origin and conservation of a unique disease-resistance gene in the grass lineage. Proc. Natl. Acad. Sci. U.S.A. 105, 1762–1767. 10.1073/pnas.0711406105 18230731 PMC2234218

[B73] Singh A. K. Sharma V. Pal A. K. Acharya V. Ahuja P. S. (2013). Genome-wide organization and expression profiling of the NAC transcription factor family in potato (*Solanum tuberosum L*.). DNA Res. 20, 403–423. 10.1093/dnares/dst019 23649897 PMC3738166

[B74] Song S. Y. Chen Y. Chen J. Dai X. Y. Zhang W. H. (2011). Physiological mechanisms underlying OsNAC5-dependent tolerance of rice plants to abiotic stress. Planta 234, 331–345. 10.1007/s00425-011-1403-2 21448719

[B75] Souer E. van Houwelingen A. Kloos D. Mol J. Koes R. (1996). The no apical meristem gene of Petunia is required for pattern formation in embryos and flowers and is expressed at meristem and primordia boundaries. Cell 85, 159–170. 10.1016/S0092-8674(00)81093-4 8612269

[B76] Sperotto R. A. Ricachenevsky F. K. Duarte G. L. Boff T. Lopes K. L. Sperb E. R.. (2009). Identification of up-regulated genes in flag leaves during rice grain filling and characterization of OsNAC5, a new ABA-dependent transcription factor. Planta 230, 985–1002. 10.1007/s00425-009-1000-9 19697058

[B77] Su H. Y. Zhang S. Z. Yin Y. L. Zhu D. Z. Han L. Y. (2015). Genome-wide analysis of NAM-ATAF1,2-CUC2 transcription factor family in *Solanum lycopersicum*. J. Plant Biochem. Biotechnol. 24, 176–183. 10.1007/s13562-014-0255-9

[B78] Su H. Zhang S. Yuan X. Chen C. Wang X. F. Hao Y. J. (2013). Genome-wide analysis and identification of stress-responsive genes of the NAM-ATAF1,2-CUC2 transcription factor family in apple. Plant Physiol. Biochem. 71, 11–21. 10.1016/j.plaphy.2013.06.022 23867599

[B79] Takada S. Hibara K. Ishida T. Tasaka M. (2001). The CUP-SHAPED COTYLEDON1 gene of *Arabidopsis* regulates shoot apical meristem formation. Development 128, 1127–1135. 11245578 10.1242/dev.128.7.1127

[B80] Takeda S. Matsuoka M. (2008). Genetic approaches to crop improvement: responding to environmental and population changes. Nat. Rev. Genet. 9, 444–457. 10.1038/nrg2342 18475268

[B81] Tang Y. Liu M. Gao S. Zhang Z. Zhao X. Zhao C.. (2012). Molecular characterization of novel TaNAC genes in wheat and overexpression of TaNAC2a confers drought tolerance in tobacco. Physiol. Plant. 144, 210–224. 10.1111/j.1399-3054.2011.01539.x 22082019

[B82] Taverner N. V. Smith J. C. Wardle F. C. (2004). Identifying transcriptional targets. Genome Biol. 5, 210. 10.1186/gb-2004-5-3-210 15005803 PMC395755

[B83] Tran L. S. Nakashima K. Sakuma Y. Simpson S. D. Fujita Y. Maruyama K.. (2004). Isolation and functional analysis of *Arabidopsis* stress-inducible NAC transcription factors that bind to a drought-responsive cis-element in the early responsive to dehydration stress 1 promoter. Plant Cell 16, 2481–2498. 10.1105/tpc.104.022699 15319476 PMC520947

[B84] Tran L. S. Nishiyama R. Yamaguchi-Shinozaki K. Shinozaki K. (2010). Potential utilization of NAC transcription factors to enhance abiotic stress tolerance in plants by biotechnological approach. GM Crops 1, 32–39. 10.4161/gmcr.1.1.10569 21912210

[B85] Udvardi M. K. Kakar K. Wandrey M. Montanari O. Murray J. Andriankaja A.. (2007). Legume transcription factors: global regulators of plant development and response to the environment. Plant Physiol. 144, 538–549. 10.1104/pp.107.098061 17556517 PMC1914172

[B86] Valliyodan B. Nguyen H. T. (2006). Understanding regulatory networks and engineering for enhanced drought tolerance in plants. Curr. Opin. Plant Biol. 9, 189–195. 10.1016/j.pbi.2006.01.019 16483835

[B87] Wang N. Zheng Y. Xin H. Fang L. Li S. (2013). Comprehensive analysis of NAC domain transcription factor gene family in *Vitis vinifera*. Plant Cell Rep. 32, 61–75. 10.1007/s00299-012-1340-y 22983198

[B88] Wu Y. Deng Z. Lai J. Zhang Y. Yang C. Yin B.. (2009). Dual function of *Arabidopsis* ATAF1 in abiotic and biotic stress responses. Cell Res. 19, 1279–1290. 10.1038/cr.2009.108 19752887

[B89] Wu Z. Xu X. Xiong W. Wu P. Chen Y. Li M.. (2015). Genome-wide analysis of the NAC gene family in physic nut (*Jatropha curcas L*.). PLoS ONE 10:e0131890. 10.1371/journal.pone.0131890 26125188 PMC4488383

[B90] Xiong L. Schumaker K. S. Zhu J. K. (2002). Cell signaling during cold, drought, and salt stress. Plant Cell 14 Suppl., S165–S183. 10.1105/tpc.000596 12045276 PMC151254

[B91] Xue G. P. Way H. M. Richardson T. Drenth J. Joyce P. A. McIntyre C. L. (2011). Overexpression of TaNAC69 leads to enhanced transcript levels of stress up-regulated genes and dehydration tolerance in bread wheat. Mol. Plant 4, 697–712. 10.1093/mp/ssr013 21459832

[B92] Yamaguchi-Shinozaki K. Shinozaki K. (2006). Transcriptional regulatory networks in cellular responses and tolerance to dehydration and cold stresses. Annu. Rev. Plant Biol. 57, 781–803. 10.1146/annurev.arplant.57.032905.105444 16669782

[B93] Yokotani N. Ichikawa T. Kondou Y. Matsui M. Hirochika H. Iwabuchi M.. (2009). Tolerance to various environmental stresses conferred by the salt-responsive rice gene ONAC063 in transgenic *Arabidopsis*. Planta 229, 1065–1075. 10.1007/s00425-009-0895-5 19225807

[B94] You J. Zhang L. Song B. Qi X. Chan Z. (2015). Systematic analysis and identification of stress-responsive genes of the NAC gene family in *Brachypodium distachyon*. PLoS ONE 10:e0122027. 10.1371/journal.pone.0122027 25815771 PMC4376915

[B95] Zheng X. Chen B. Lu G. Han B. (2009). Overexpression of a NAC transcription factor enhances rice drought and salt tolerance. Biochem. Biophys. Res. Commun. 379, 985–989. 10.1016/j.bbrc.2008.12.163 19135985

[B96] Zhong R. Lee C. Ye Z. H. (2010). Global analysis of direct targets of secondary wall NAC master switches in *Arabidopsis*. Mol. Plant 3, 1087–1103. 10.1093/mp/ssq062 20935069

